# Diagnostic Accuracy of Stool Xpert MTB/RIF for Detection of Pulmonary Tuberculosis in Children: a Systematic Review and Meta-analysis

**DOI:** 10.1128/JCM.02057-18

**Published:** 2019-05-24

**Authors:** Emily MacLean, Giorgia Sulis, Claudia M. Denkinger, James C. Johnston, Madhukar Pai, Faiz Ahmad Khan

**Affiliations:** a Department of Epidemiology, Biostatistics, and Occupational Health, McGill University, Montreal, Canada; b McGill International TB Centre, McGill University, Montreal, Canada; c Foundation for Innovative New Diagnostics, Geneva, Switzerland; d Department of Infectious Diseases, University of Heidelberg, Heidelberg, Germany; e Division of Respiratory Medicine, University of British Columbia, Vancouver, Canada; f BC Centre for Disease Control, Vancouver, Canada; g Respiratory Epidemiology and Clinical Research Unit, Research Institute of the McGill University Health Centre and Montreal Chest Institute, Montreal, Canada; Washington University School of Medicine

**Keywords:** Xpert MTB/RIF assay, childhood TB, pediatric infectious disease, pulmonary tuberculosis, stool

## Abstract

Invasive collection methods are often required to obtain samples for the microbiological evaluation of children with presumptive pulmonary tuberculosis (PTB). Nucleic acid amplification testing of easier-to-collect stool samples could be a noninvasive method of diagnosing PTB. We conducted a systematic review and meta-analysis to evaluate the diagnostic accuracy of testing stool with the Xpert MTB/RIF assay (“stool Xpert”) for childhood PTB. Four databases were searched for publications from January 2008 to June 2018. Studies assessing the diagnostic accuracy among children of stool Xpert compared to a microbiological reference standard of conventional specimens tested by mycobacterial culture or Xpert were eligible. Bivariate random-effects meta-analyses were performed to calculate pooled sensitivity and specificity of stool Xpert against the reference standard. From 1,589 citations, 9 studies (*n* = 1,681) were included. Median participant ages ranged from 1.3 to 10.6 years. Protocols for stool processing and testing varied substantially, with differences in reagents and methods of homogenization and filtering. Against the microbiological reference standard, the pooled sensitivity and specificity of stool Xpert were 67% (95% confidence interval [CI], 52 to 79%) and 99% (95% CI, 98 to 99%), respectively. Sensitivity was higher among children with HIV (79% [95% CI, 68 to 87%] versus 60% [95% CI, 44 to 74%] among HIV-uninfected children). Heterogeneity was high. Data were insufficient for subgroup analyses among children under the age of 5 years, the most relevant target population. Stool Xpert could be a noninvasive method of ruling in PTB in children, particularly those with HIV. However, studies focused on children under 5 years of age are needed, and generalizability of the evidence is limited by the lack of standardized stool preparation and testing protocols.

## Abstract (precis)

Invasive collection methods are often required to obtain samples for the microbiological evaluation of children with presumptive pulmonary tuberculosis (PTB). Nucleic acid amplification testing of easier-to-collect stool samples could be a noninvasive method of diagnosing PTB.

## INTRODUCTION

At least 1 million incident tuberculosis (TB) cases and 230,000 TB-related deaths are estimated to have occurred among children in 2017, accounting for approximately 10% of total cases and 15% of deaths (1). Pulmonary TB (PTB) is the most common form of childhood TB (2). Xpert MTB/RIF (Xpert) (Cepheid, USA), an automated cartridge-based PCR assay, is currently recommended by the World Health Organization (WHO) as the initial diagnostic test in presumptive PTB cases for adults and children (3). Minimal sample preparation is required, and test results are produced within 2 h. In a meta-analysis that pooled data from sputum smear-positive and -negative subjects, the performance of Xpert on respiratory samples had a sensitivity of 62% (95% credible interval, 51 to 73%) and a specificity of 98% (95% credible interval, 97 to 99%). The use of Xpert on sputum is thus more sensitive than smear microscopy. Moreover, Xpert has several operational advantages over mycobacterial culture, the gold standard for TB diagnosis (4). However, in children under 5 years old, and particularly in those under 2 years old, the collection of sputum specimens is difficult and often requires invasive methods that are challenging to implement in resource-limited settings (e.g., nasopharyngeal/nasogastric aspiration or bronchoscopy) and not widely available (2). Furthermore, as pediatric TB is typically paucibacillary, the sensitivity of currently deployed tests is diminished in children versus adults (5).

Mycobacterium tuberculosis-containing sputum may be swallowed, particularly during sleep, and acid-fast bacilli have been shown to survive digestion and are detectable in stool (6, 7). As such, stool may represent a more acceptable and feasible alternative to conventional specimens for the evaluation of suspected childhood PTB. The use of Xpert on stool has not been included in recommendations by the WHO, nor has any claim been made by the manufacturer regarding stool. However, several groups have now developed preprocessing methods in order to use Xpert on stool for the diagnosis of childhood TB.

We performed a systematic review and meta-analysis of the diagnostic performance of Xpert using stool samples for PTB in children.

## MATERIALS AND METHODS

### Protocol and registration.

The protocol for this systematic review and meta-analysis was registered at the International Prospective Register of Systematic Reviews (PROSPERO) (identifier CRD42017079836).

### Search strategy and information sources.

PubMed, EMBASE, Scopus, and the Cochrane Library were systematically searched from 1 January 2008 until 15 June 2018. The search strategy was developed with a medical librarian and based on key validated terms for “children” and “Xpert,” as well as “tuberculosis,” with no filters applied. The full search strategies for each database are presented in Text S1 in the supplemental material. Experts in TB diagnostics were consulted to identify relevant papers that may have been missed by the search strategy. Citations of reviews and included publications were also searched.

### Eligibility criteria.

Publications in English, French, Italian, Mandarin, Spanish, and Portuguese; of any design and sampling strategy; and of any enrollment timing (prospective, retrospective, or cross-sectional) were eligible for inclusion. Conference proceedings and abstracts, commentaries, editorials, and reviews were excluded, as were studies with a sample size of less than 10. To be included, eligible studies must have reported the diagnostic performance of stool Xpert in patients under 16 years old, compared to a microbiological reference standard for the diagnosis of PTB. Studies that did not explicitly state that their focus was PTB were eligible if the types of specimens used for the reference standard were those that are typically used for PTB diagnosis (e.g., gastric aspirate). Studies that used banked sputum and stool specimens originally collected from children were also eligible.

### Study screening and selection.

Search results were imported into a citation manager, and duplicates were removed. Two authors (E. MacLean and G. Sulis) independently screened citations by title and abstract per predefined eligibility criteria, followed by full-text review for all selected studies. Results disagreed upon were discussed, and a third reviewer consulted if necessary (F. Ahmad Khan).

### Data extraction.

A data extraction form was piloted by two reviewers (E. MacLean and G. Sulis) with critical input from a third (C. M. Denkinger). Two reviewers (E. MacLean and G. Sulis) independently extracted results from all included studies using a standardized form (Text S2). After data extraction, results were compared, and disagreements were discussed until a consensus was reached. Study authors were contacted for missing performance data, clarification regarding reference standard definitions, and sample preparation techniques. Using these data and figures indicated in the publications, we reconstructed two-by-two tables for stool Xpert performance compared to the microbiological reference standard and, where applicable, the clinical reference standard.

### Risk-of-bias assessment.

The Quality Assessment of Diagnostic Accuracy Studies 2 (QUADAS-2) tool (8) was used to assess each included study’s risk of bias. No formal assessment of publication bias was made, as traditional methods such as funnel plots and regression tests are not helpful for diagnostic studies (9).

### Reference standards.

Acceptable microbiological reference standards were mycobacterial culture or Xpert MTB/RIF, performed on specimens that are conventionally used to diagnose childhood PTB (nasogastric aspirates, gastric lavage fluid, nasopharyngeal aspirates, and expectorated sputum). No studies included stool mycobacterial culture in their diagnostic workup. Stool Xpert was not included in the reference standard.

Childhood PTB is often clinically diagnosed (i.e., without microbiological confirmation). As such, we also examined the performance of stool Xpert compared to clinical reference standards that are compatible with updated international guidelines (5). Studies that followed these guidelines used a combination of signs and symptoms, chest radiography, epidemiological history, and tuberculin skin test (TST) results to classify children as “likely TB,” “unconfirmed TB,” and “unlikely TB” (Table S1). For our purposes, we dichotomized these outcomes into “likely/possible TB” and “unlikely TB.”

### Statistical analysis.

Data from reconstructed two-by-two tables were used to calculate sensitivity and specificity and the associated 95% confidence intervals (CIs). In cases of empty cells in two-by-two tables, a zero correction was made by replacing the cell with 0.5. Aggregate-data meta-analyses were performed with bivariate random-effect hierarchical models (10) to estimate pooled sensitivity and specificity for stool Xpert compared to the microbiological reference standard and, separately, compared to the clinical reference standard. We also estimated pooled sensitivity and specificity stratified by HIV status. Results from individual studies and pooled estimates are presented on forest plots. To assess between-study heterogeneity, we used the *I*^2^ statistic (11). In a sensitivity analysis, we estimated pooled sensitivity and specificity after excluding studies that used Xpert MTB/RIF but not mycobacterial culture of conventional specimens as the microbiological reference standard. All analyses were conducted using the Midas package in STATA (STATA 15; Stata Corp., USA) (12). The study is reported according to PRISMA guidelines (Table S2) (13).

## RESULTS

### Search results.

**FIG 1:**
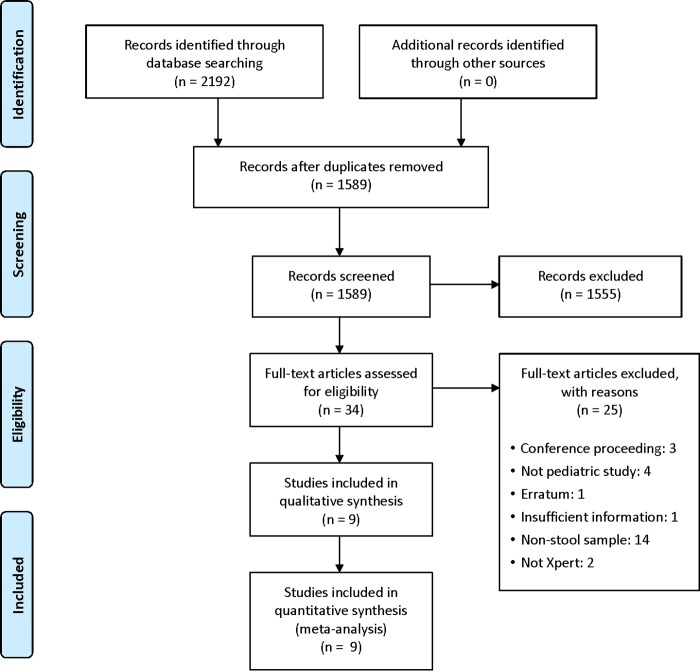
PRISMA study flow diagram.

Our search identified 1,589 unique citations from which 34 studies were selected for full-text review, and 9 studies met inclusion criteria (Fig. 1).

### Study and participant characteristics.

Study and patient characteristics are presented in Table 1. Among the 9 studies that we included, African countries were most well represented (7/9), whereas 2 studies recruited participants from Asia. One study had multiple sites across two continents, whereas the others were single-country studies. In total, 1,681 children from 9 studies were included in our meta-analysis of stool Xpert’s diagnostic performance compared to a microbiological reference standard, and 869 children from 5 studies were included in the comparison against a clinical reference standard. The prevalence of microbiologically confirmed cases per study ranged widely, from 2.6% (14) to 54% (15). The prevalence of clinically confirmed or unconfirmed cases was much higher, ranging from 35% (16) to 100% (17). Table S1 in the supplemental material provides details on clinical reference standard definitions of the included studies.

**TABLE 1:** Features of included studies and participants^*g*^

Study authors, yr (reference)	Location(s)	No. of eligible children	Age range, median (yr) (IQR)	No. of patients/total no. of patients (%)					Clinical features reported	EPTB status (no. of patients with EPTB [%])	Reference standard	Sample type(s) used for reference standard	Total no. of specimens included in analysis	No. of microbiologically confirmed cases (%)	No. of clinically confirmed/unconfirmed cases (%)	No. of cases of clinically unlikely TB (%)	No. of contaminated cultures (%)
				Female	TB history	TB contact history	TST positive	HIV positive									
Banada et al., 2016 (15)	South Africa	40	0–15, NR	21/38 (55)	NR	16/38 (42)	NR	16/38 (42)	Cough, EP symptoms, wt loss	PTB only	Xpert	IS, GA	37	20 (54)			NR
Chipinduro et al., 2017 (17)	Zimbabwe	218	5–16, 10.6 (8–13)	123/218 (56)	17/218 (7.8)	51/218 (23)	NR	111/198 (56)	Cough, wt loss, night sweats, fever, appetite loss	PTB only^*a*^	Culture^*c*^/Xpert	IS	218	19 (8.7)			NR
											CRS^*b*^		32		32 (100)	0 (0)	NR
Hasan et al., 2017 (16)	Pakistan	50	0–15, 6.8 (2–9)	22/50 (44)	NR	27/50 (54)	NR	0/50 (0)	Cough, EP symptoms, wt loss	PTB only	Culture^*d*^/Xpert	Sputum, GA	49	11 (22)			NR
											CRS^*b*^		49		17 (35)	32 (65)	NR
LaCourse et al., 2018 (18)	Kenya	165	0–12, 2 (1.1–4.8)	75/165 (45)	NR	20/162 (12)	7/151 (4.6)	165/165 (100)	Cough, lethargy, fever, failure to thrive	PTB only^*a*^	Culture^*f*^/Xpert	Sputum, GA	147	11 (7.5)			NR
											CRS^*b*^		165		85 (52)	80 (48)	NR
Marcy et al., 2016 (20)	Burkina Faso, Cambodia, Cameroon, Vietnam	272	0–13, 7.2 (4.1–7.2)	132/272 (49)	49/272 (18)	58/272 (21)	50/272 (18)	272/272 (100)	Cough, wt loss, lethargy, fever, broad-spectrum Abx failure, CXR abnormality	PTB only^*a*^	Culture^*e*^	GA, IS, NS, string	272	27 (10)			NR
											CRS^*b*^		272		245 (90)	27 (10)	NR
Moussa et al., 2016 (21)	Egypt	115	1–16, NR	45/115 (39)	NR	29/115 (25)	13/67 (19)	0/115 (0)	Cough, wt loss, night sweats, fever, CXR abnormality	PTB only	Culture^*c*^	Sputum, IS	115	36 (31)			0/115 (0)
Nicol et al., 2013 (22)	South Africa	115	1–15, 2.6 (1.6–4.8)	NR	0/115 (0)	NR	NR	17/115 (15)	Cough, wt loss, CXR abnormality	PTB only	Culture^*d*^	IS	115	17 (15)			NR
Orikiriza et al., 2018 (14)	Uganda	357	1–14, NR	178/392 (45)	8/392 (2.0)	76/391 (19)	99/383 (26)	121/388 (31)	Cough, wt loss, night sweats, lethargy, fever	PTB only^*a*^	Culture^*e*^/Xpert	Sputum, IS	349	9 (2.6)			6/357 (1.7)
Walters et al., 2017 (19)	South Africa	379	0–13, 1.3 (0.8–2.4)	184/379 (49)	27/379 (7.1)	214/379 (56)	82/294 (28)	51/379 (13)	Cough, wt loss, fever	Mix of EPTB and PTB (35/379 [9.2])	Culture^*d*^/Xpert	GA, IS, NA, string	379	72 (19)			NR
											CRS^*b*^		351		242 (69)	109 (31)	NR

a Implied only pulmonary TB cases based on collection of respiratory samples only.

b Definitions of each clinical reference standard are given in Table S1 in the supplemental material.

c Lowenstein-Jensen solid culture.

d Bactec MGIT liquid culture.

e Both Lowenstein-Jensen solid cultures and MGIT liquid culture.

f MGIT liquid culture, with positive samples then being subcultured on Lowenstein-Jensen medium for 3 additional weeks.

g Some studies included separate comparisons of stool Xpert for microbiological and clinical reference standards. Abbreviations: Abx, antibiotics; CRS, clinical reference standard; CXR, chest X ray; EP, extrapulmonary; EPTB, extrapulmonary TB; GA, gastric aspirate; IQR, interquartile range; IS, induced sputum; NA, nasopharyngeal aspirate; NR, not reported; TST, tuberculin skin test.

Studies enrolled children from 0 to 16 years of age. The ratio of females to males was generally balanced. The percentage of participants with a documented history of TB disease contact, when reported (5/9 studies), ranged from 12% (18) to 56% (19). Most studies did not include information about tuberculin skin test (TST) results. Two studies included only children with HIV (18, 20), and two restricted enrollment to HIV-negative children (16, 21); the remainder had a mixed population.

### Sample processing.

Table 2 shows the sample preparation steps utilized in each study. In one study (19), two sample preparation methods were attempted, with results ultimately being pooled. Most studies (6/9) obtained one stool sample from enrolled children, typically within 24 h of obtaining respiratory samples. Samples were either used immediately or stored for later use, except for one study (20) which used some samples immediately and some after freezing and a second study (19) which stored samples collected at the child’s home and immediately used those collected at the health care center. As information on sample storage was not available for all studies, subgroup analysis could not be performed per sample storage method.

**TABLE 2:** Details of stool sample storage and processing for each of the included studies^*a*^

Study authors, yr (reference)	No. of samples collected, mass (g)	Stool sample collection timing	Immediate use	Storage method	Stool mass used for Xpert (g)	First reagent(s) added to stool	Homogenization method(s)	Duration of specimen settling	Additional reagent(s) and or filtering/processing procedure(s)	Pellet processing procedure	Final sample loaded into cartridge
Banada et al., 2016 (15)	1, 5	NR	No	4°C for 7 days	0.6	2 ml processing buffer (AL buffer, 10% povidone), 2 ml Xpert buffer	Vortexing with glass beads	30 min at RT	All syringe filtered	No pellet	2 ml added to cartridge
Chipinduro et al., 2017 (17)	1, 5	Within 24 h of respiratory sample collection	No	4°C for max of 2 days	0.15, using sterile loop	2.4 ml PBS	Vortexing	20 min at RT	1 ml supernatant taken, centrifuged at 3,200 rpm for 15 min	Pellet resuspended in 1 ml PBS	Diluted 2:1 in buffer, added to cartridge
Hasan et al., 2017 (16)	1, NR	Within 24 h of respiratory sample collection	No	2–8°C (days NR), taken to tertiary hospital, stored at −80°C	0.15	2.4 ml PBS	Vortexing	20 min at RT	1 ml supernatant taken, centrifuged at 3,500 rpm for 15 min	Pellet resuspended in 1 ml PBS	Diluted 2:1 in buffer, added to cartridge
LaCourse et al., 2018 (18)	1, 2–15	Within 24 h of respiratory sample collection	Yes	NA	NR	Equal vol of PBS	Manual homogenization	12 to 48 h at 2–5°C	All filtered through fine filter, vortexed; added to equal vol of NaOH-NALC; PBS (concn NR) added to 40 ml and centrifuged twice	Pellet resuspended in 1.4 ml PBS by vortexing	Diluted 2:1 in buffer, added to cartridge
Marcy et al., 2016 (20)	NR, 0.5	NR	Both	Some frozen (temp and days NR)	0.5	10 ml Sheather’s solution (28% sucrose)	Manual homogenization, vortexing for 30 s	NR	All filtered through funnel gauze; centrifuged at 100 × *g* for 1 min	No pellet	0.5 ml supernatant, 1.8 ml buffer added to cartridge; mixture allowed to sit for 15 min at RT; shaken; run
Moussa et al., 2016 (21)	2, 2	NR	Yes	NA	2	10 ml distilled H_2_O	Vortexing	NR	Supernatant (concn NR) taken, centrifuged at 4,000 rpm for 20 min	Pellet decontaminated in 10 ml 3% NALC-NaOH for 15 min at RT; added to 40 ml PBS; centrifuged for 20 min; pellet resuspended in 1 ml PBS	Diluted 2:1 in buffer, added to cartridge
Nicol et al., 2013 (22)	1, NR	At baseline	No	−80°C within 2 h for max of 6 mo	0.15 using FLOQ swabs	2.4 ml PBS	Vortexing	20 min at RT	1 ml supernatant taken, centrifuged at 3,200 rpm for 15 min	Pellet resuspended in 1 ml PBS	Diluted 2:1 in buffer, added to cartridge
Orikiriza et al., 2018 (14)	1, NR	NR	Yes	NA	NR	Saline solution	Vortexing	5 min at RT	5 ml mixture taken, added to NaOH-NALC, vortexed, with standing for 20 min; PBS added to 50 ml and centrifuged at 3,000 × *g* for 20 min at 4°C	Pellet decontaminated with NaOH-NALC method, respun; pellet resuspended in 1.5 ml unspecified buffer	0.5 ml added to cartridge
Walters et al., 2017 (19)	1, 0.3–5	Within 7 days of respiratory sample collection	Both	2–8°C for max of 3 days if collected at home	<5	20 ml PBS	Vortexing	None	5 ml mixture taken, added to NALC-NaOH	Concentration	Diluted 2:1 in buffer, added to cartridge
	1, 0.3–5	Within 7 days of respiratory sample collection	Both	2–8°C for max of 3 days if collected at home	1–4	10 ml PBS	Vortexing	None	All centrifuged at 3,000 × *g* at 4°C for 20 min	Pellet resuspended in 10 ml by vortexing for 20 s; centrifuged at 2,000 × *g* for 1 s; supernatant kept	1 ml supernatant added to cartridge

a Abbreviations: max, maximum; NA, not applicable; NR, not reported; PBS, phosphate-buffered saline; RT, room temperature; NALC-NaOH, *N*-acetyl-l-cysteine–sodium hydroxide.

The mass of stool utilized, and its collection method, varied: 0.15 g of bulk stool (16), 0.15 g using a sterile loop (17), a flocked rectal swab (22), 0.5 g (21), 0.6 g (15), 2 g (20), and 5 g (19). A diluent solution, such as phosphate-buffered saline (PBS), distilled water, or a sucrose solution, was added to the stool before homogenization, in various quantities, typically followed by vortexing. Most studies (6/9) reported a period of sample settling before further workup. Final sample preparation methods were quite varied but included either centrifugation or filtering through a syringe filter or gauze, primarily to remove large particles, before final addition of the sample to the Xpert cartridge (Table 2).

### Quality assessment.

**FIG 2:**
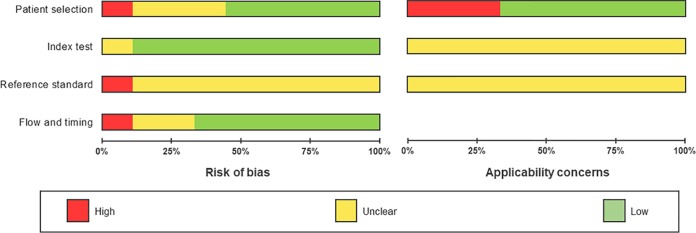
QUADAS-2 risk of bias and applicability concerns graph. The review authors’ judgements about each domain are presented as percentages across the 9 included studies.

Figure 2 displays the overall risk of bias and applicability concerns of the 9 studies included in our meta-analysis. Figure S1 presents the individual studies’ quality assessment results. In the patient selection domain (Fig. 2), five studies were at low risk of bias, and one study (15) was at high risk of bias due to its use of a case-control design, whereas the remaining eight were either cross-sectional or cohort studies. Risk of bias was high for one study because of convenience sampling (16) and unclear in two studies because of an unclear sampling strategy and inappropriate exclusions of certain children (17, 21). With respect to applicability, the majority of studies (Table 1) included children who presented with symptoms suggestive of TB. Two studies (18, 20) included only children with HIV, and because it is known that Xpert performs differentially for those who are HIV infected (23), these studies were scored for applicability concerns as high. One study (15) tested only samples from confirmed TB cases and noncases, which does not represent a typical clinical scenario, so we also rated applicability concerns as high.

The conduct of the index test generally was at low risk of bias, as Xpert is an automated assay with a predefined cutoff of detection that produces a binary response. However, since there is no standardized operating protocol for stool samples and no internationally recommended procedure for sample storage and processing, applicability concerns regarding the index test’s conduct are unclear (Fig. 2).

In light of the inherent limitations of microbiological tests for diagnosing childhood PTB, we classified 8/9 studies as having an unclear risk of bias with respect to correctly classifying the target condition despite having used culture as the reference test. The exception was one study that was scored as having a high risk of bias as its microbiological reference standard did not include culture. Both culture and Xpert are automated assays, so we scored the risk of bias as low regarding test result interpretation. Additionally, all studies’ reference standards were performed in regional or central reference laboratories, so we expect bias from operator error to be of low concern. Applicability concerns were uniformly unclear.

We scored the risk of bias as low for all studies with respect to the appropriateness of the time interval between the index test and the reference standard, as all studies reported running stool Xpert within 7 days of specimen collection (Fig. 2).

### Meta-analysis of diagnostic accuracy.

**FIG 3:**
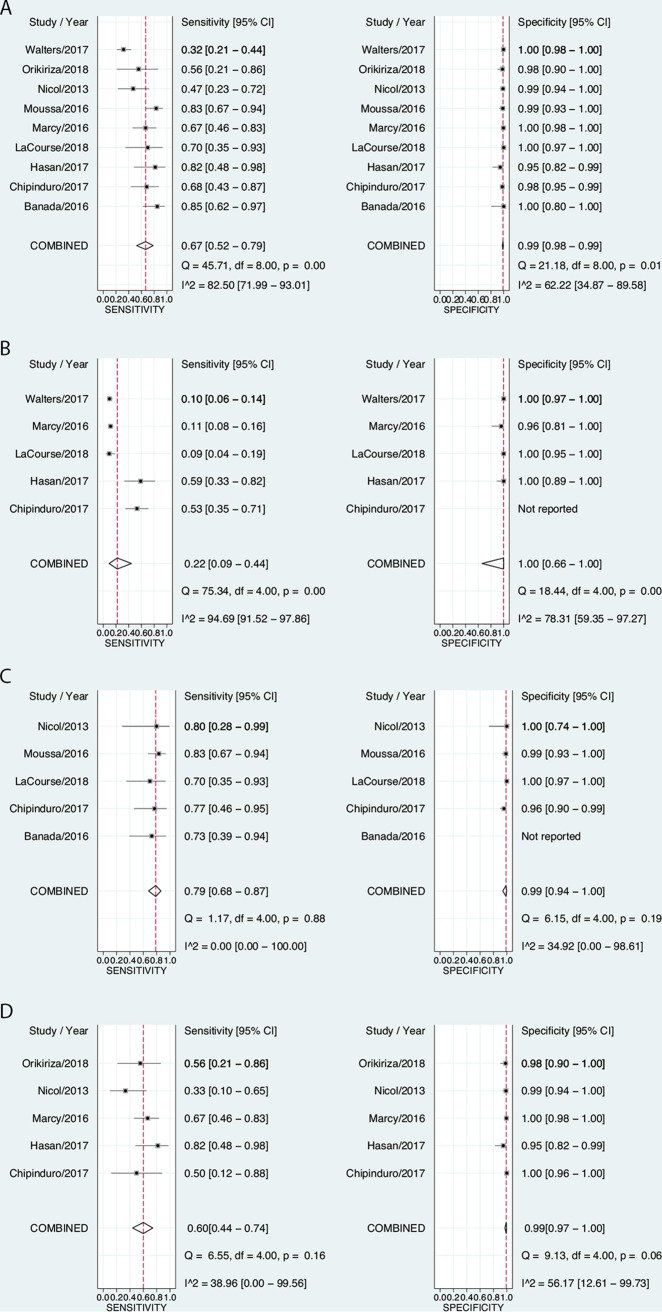
(A) Forest plots of stool Xpert’s diagnostic performance compared to a microbiological reference standard of culture or Xpert positivity on respiratory samples (14–22). Two studies (18, 20) presented results from “intention-to-treat” (ITT) analyses, where any child who produced any sample was included, as well as “per-protocol” analyses, where only children who produced all requested samples were included. In these instances, we meta-analyzed the ITT results to avoid selection bias. (B) Forest plots of stool Xpert’s diagnostic performance compared to a clinical reference standard of “likely/possible TB” or “unlikely TB.” (C) Forest plots of diagnostic performance of stool Xpert in children with HIV compared to a microbiological reference standard. (D) Forest plots of diagnostic performance of stool Xpert in HIV-negative children compared to a microbiological reference standard.

For comparison against the microbiological reference standard, sensitivities of stool Xpert varied from 32% (19) to 85% (15), while specificity was uniformly very high (Fig. 3A). The pooled sensitivity was 67% (95% CI, 52 to 79%), and the pooled specificity was 99% (95% CI, 98 to 99%). *I*^2^ values for sensitivity and specificity were 83% (95% CI, 72 to 93%) and 62% (95% CI, 35 to 90%), respectively, indicating high between-study heterogeneity, particularly for sensitivity. For the clinical reference standard comparison, the pooled sensitivity of stool Xpert was 22% (95% CI, 9.0 to 44%), while the specificity was 100% (95% CI, 66 to 100%) (Fig. 3B).

Although 7/9 studies included children with HIV, only 5/9 studies provided sufficient information to construct two-by-two tables (15, 17, 18, 21, 22) (2 of these studies enrolled only children with HIV [18, 21]) (Fig. 3C). One study (15) did not provide sufficient information to calculate specificity among children with HIV. Data from children who were HIV negative were available from 5 studies (14, 16, 17, 20, 22) (Fig. 3D). Using the microbiological reference standard, among children with HIV, the sensitivity of stool Xpert was 79% (95% CI, 68 to 87%), and the pooled specificity was 99% (95% CI, 94 to 100%) (Fig. 3C); among those without HIV, the sensitivity was 60% (95% CI, 44 to 79%), and the specificity was 99% (95% CI, 97 to 100%) (Fig. 3D). For both sensitivity and specificity, *I*^2^ values were lower in HIV-stratified analyses than when data from all studies were pooled (Table 3), suggesting that HIV partially explained the between-study heterogeneity.

**TABLE 3:** Results of meta-analyses for estimated stool Xpert sensitivity and specificity^*a*^

Comparison	Main results			Results of sensitivity analysis excluding the study that did not use culture as reference standard		
	No. of studies included (no. of children included)	Pooled sensitivity (%) (95% CI); *I*^2^ statistic (95% CI)	Pooled specificity (%) (95% CI); *I*^2^ statistic (95% CI)	No. of studies included (no. of children included)	Pooled sensitivity (%) (95% CI); *I*^2^ statistic (95% CI)	Pooled specificity (%) (95% CI); *I*^2^ statistic (95% CI)
Stool Xpert against microbiological reference standard	9^*b*^ (1,681)	67 (52–79); 83 (72–93)	99 (98–99); 62 (35–90)	8^*f*^ (1,644)	64 (49–76); 81 (69–93)	99 (98–100); 61 (31–91)
Stool Xpert against clinical reference standard	5^*c*^ (869)	22 (9.0–44); 95 (92–98)	100 (66–100); 78 (59–97)	Not applicable	Not applicable	Not applicable
Stool Xpert against microbiological reference standard for children with HIV	5^*d*^ (395)	79 (68–87); 0 (0–100)	99 (94–100); 35 (0–99)	5^*g*^ (379)	80 (68–88); 0 (0–100)	99 (94–100); 51 (0–100)
Stool Xpert against microbiological reference standard for HIV-negative children	7^*e*^ (974)	61 (40–79); 39 (0–100)	99 (98–100); 56 (13–100)	Not applicable	Not applicable	Not applicable

a The *I*^2^ statistic was used to quantify the effect of between-study heterogeneity.

b From references 14–22.

c From references 16–20.

d From references 15, 17, 18, 21, and 22.

e From references 14–17, 19, 20, and 22.

f From references 14 and 16–22.

g From references 17, 18, 21, and 22.

Results of the sensitivity analysis in which we excluded the study that did not use mycobacterial culture as part of the reference standard (15) are presented in Fig. S2. Pooled sensitivity and specificity estimates combining data from all studies and data stratified by HIV status were all similar to those estimated in our main analyses, as was between-study heterogeneity. Pooled estimates from our main analysis and from this sensitivity analysis are summarized in Table 3.

We undertook two *post hoc* sensitivity analyses. In the first, we sought to determine whether the quantity of stool used for testing was associated with diagnostic accuracy (assuming that a higher mass might increase sensitivity). There were too few studies to estimate pooled accuracy stratified by stool mass used; however, visual inspection of forest plots showed no obvious trend to support a minimum quantity (Fig. S3). In the second sensitivity analysis, we evaluated whether the burden of TB in the country where a study was conducted was associated with the accuracy of stool Xpert. As shown in Fig. S4, there was no clear trend to suggest such an association.

## DISCUSSION

In this systematic review and meta-analysis, we found that the sensitivity and specificity of stool Xpert (67% [95% CI, 52 to 79%] and 99% [95% CI, 98 to 99%], respectively) for the diagnosis of microbiologically confirmed childhood PTB were comparable to what has been reported for the performance of Xpert on respiratory specimens (62% [95% credible interval, 51 to 73%] and 98% [95% credible interval, 97 to 99%], respectively) (4). Sensitivity and specificity varied by HIV status. As stool collection is noninvasive, this is of substantial interest for the medical evaluation of children with suspected PTB, but a number of limitations of the existing evidence highlight the need for more research, and greater standardization of testing, before policy formulation.

Among the most important limitations of the evidence base is the lack of data on performance in the subpopulation of children for whom stool Xpert is of greatest potential clinical utility, those under the age of 5 years, and especially the subgroup under the age of 2 years. Only one study compared accuracy between age categories, and a cutoff of 10 years of age was used (17).

We observed substantial between-study heterogeneity in diagnostic accuracy, mostly for sensitivity. Different approaches to participant selection likely contributed to this, in particular the use of a case-control design (15) and nonconsecutive sampling (16, 21), which are at a higher risk of introducing bias into a study. Data also suggested that heterogeneity was partly explained by differences in the prevalence of HIV infection. The higher sensitivity of stool Xpert among children with HIV has also been observed for other specimen types in this population (4, 24), perhaps as a result of more severe TB disease in HIV-TB-coinfected children.

We found substantial variability in protocols for performing stool Xpert, with each study taking a unique approach. Differences were seen at all steps: (i) at stool collection, different methods of sampling, numbers of specimens, and volumes of stool were used; (ii) different reagents were added to stool samples before homogenization, and all studies utilized different additional reagents; and (iii) dissimilar filtration methods and decontamination steps were adopted. Future studies should ensure, at minimum, complete reporting of protocols for stool collection processing and testing. A standardized protocol would be of value, as would a standardized stool collection-and-processing kit.

Our systematic review and meta-analysis has a number of strengths. First, all included studies reported using a microbiological reference standard for comparison to stool Xpert, and 8 out of 9 studies used liquid or solid culture. While the imperfect nature of any reference standard for diagnosing pediatric TB means that the true number of affected children is always unknown, the accuracy of stool Xpert against microbiological confirmation is likely a closer estimation of its true accuracy than its performance compared to the clinical reference standard (as symptoms of PTB are nonspecific). Second, by systematically assessing each study’s sample preparation and processing techniques, we found substantial variability in methods of performing stool Xpert and were also able to identify obstacles to implementation. For example, most protocols required at least one centrifugation step, which is inauspicious in terms of translating this assay to a lower health care system level. Finally, we utilized a sensitive and validated search strategy that covered six languages.

The present work also has some limitations. First, data were insufficient, and there were too few studies for us to perform stratified or metaregression analyses to assess most demographic-related potential causes of observed heterogeneity. Hence, we suggest that in addition to HIV-stratified results, future studies of stool Xpert should also ensure that reporting is stratified by age, gender, and extent of radiographic disease. Second, while we identified wide variability in sampling and stool processing, we could not explore these as sources of heterogeneity or determine if any processing workflows were potentially superior. Third, we did not include one study concerning the performance of stool Xpert on samples from children (25) that was reported after our systematic search was completed and therefore was not included in our meta-analysis. However, including it in our pooled analyses did not significantly alter sensitivity or specificity estimates (see Fig. S5 in the supplemental material). Finally, our pooled estimates came from study populations with a high prevalence of TB; hence, it is possible that these estimates may not be generalizable to settings of lower TB burdens.

Given that these preliminary studies of stool Xpert suggest high specificity and moderate sensitivity, its potential role in the diagnostic pathway would be as a first-line rule-in test rather than as a triage test to rule out PTB. Studies assessing whether stool Xpert has value as an add-on test in combination with currently deployed assays will be useful, as will studies assessing the effect of repeat testing on sensitivity.

### Conclusion.

Preliminary data suggest that the use of Xpert on stool specimens may be potentially useful as a rule-in test, but a standardized stool sample preparation protocol is lacking, and the accuracy of stool Xpert in children under 5 years old, the subgroup for whom the test could bring the most added value, remains largely unknown.

## Supplementary Material

Supplemental file 1
