# Supplementary material for: Diagnostic Accuracy of Stool Xpert MTB/RIF for Detection of Pulmonary Tuberculosis in Children: a Systematic Review and Meta-analysis
Source: J Clin Microbiol. 2019 May 24;57(6):e02057-18. doi: 10.1128/JCM.02057-18 (PMC6535592; doi:10.1128/JCM.02057-18)
Supplement: Supplemental file 1 [file JCM.02057-18-s0001.pdf]

Supplementary information

Diagnostic accuracy of stool Xpert MTB/RIF for the detection of pulmonary tuberculosis in children: a systematic review and meta-analysis

Emily MacLean, Giorgia Sulis, Claudia M. Denkinger, James C. Johnston, Madhukar Pai, Faiz Ahmad Khan

Supplementary Information contents

Table S1: Clinical reference standard defintions

Table S2: PRISMA checklist

Figure S1: QUADAS risk of bias and applicability concerns

Figure S2a: Forest plots of sensitivity analysis of stool Xpert's diagnostic performance compared to a microbiological reference standard of culture only on respiratory samples using 8 studies

Figure S2b: Forest plots of sensitivity analysis of stool Xpert's diagnostic performance in children living with HIV compared to a microbiological reference standard of culture only on respiratory samples using 8 studies

Figure S3: Forest plots of stool Xpert performance arranged by sample mass

Figure S4: Forest plots of stool Xpert's diagnostic performance arranged by study countries' TB burden

Figure S5: Forest plots of stool Xpert's diagnostic performance compared to a microbiological reference standard of culture or Xpert positivity on respiratory samples using 10 studies

Text S1: Search strategies employed

Text S2: Information extracted from each included study

References

Study	Clinical reference standards	Notes
Chipinduro 2017 (1)	International guidelines (bacteriological confirmation, symptoms, contact history or immunologic evidence, treatment response) (2)	Chest x-ray not uniformly available to all presenting children
Hasan 2017 (3)	Modified Kenneth-Jones criteria (BCG vaccine and scar, contact history, measles history, chest x-ray, PCM grade 3, immunocompromised/immunosuppressed) (4)	Algorithm utilized by Pakistan Pediatric Association
LaCourse 2018 (5)	International guidelines (bacteriological confirmation, symptoms, chest x-ray, contact history or immunologic evidence, treatment response) (2)	-
Marcy 2016 (6)	International guidelines (bacteriological confirmation, symptoms, chest x-ray, contact history or immunologic evidence, treatment response) (2)	-
Walters 2017 (7)	International guidelines (bacteriological confirmation, symptoms, chest x-ray, contact history or immunologic evidence, treatment response) (2)	Decision to administer treatment not based on research case definitions

Table S1: **Clinical reference standard definitions used by publications included in meta-analysis for "confirmed/unconfirmed" TB.**

PRISMA-DTA Checklist

Section/topic	#	PRISMA-DTA Checklist Item	Reported on page #
**TITLE / ABSTRACT**			
Title	1	Identify the report as a systematic review (+/- meta-analysis) of diagnostic test accuracy (DTA) studies.	1
Abstract	2	Abstract: See PRISMA-DTA for abstracts.	2-3
**INTRODUCTION**			
Rationale	3	Describe the rationale for the review in the context of what is already known.	4
Clinical role of index test	D1	State the scientific and clinical background, including the intended use and clinical role of the index test, and if applicable, the rationale for minimally acceptable test accuracy (or minimum difference in accuracy for comparative design).	4
Objectives	4	Provide an explicit statement of question(s) being addressed in terms of participants, index test(s), and target condition(s).	5
**METHODS**			
Protocol and registration	5	Indicate if a review protocol exists, if and where it can be accessed (e.g., Web address), and, if available, provide registration information including registration number.	5
Eligibility criteria	6	Specify study characteristics (participants, setting, index test(s), reference standard(s), target condition(s), and study design) and report characteristics (e.g., years considered, language, publication status) used as criteria for eligibility, giving rationale.	5-6
Information sources	7	Describe all information sources (e.g., databases with dates of coverage, contact with study authors to identify additional studies) in the search and date last searched.	5
Search	8	Present full search strategies for all electronic databases and other sources searched, including any limits used, such that they could be repeated.	Text S1
Study selection	9	State the process for selecting studies (i.e., screening, eligibility, included in systematic review, and, if applicable, included in the meta-analysis).	6
Data collection process	10	Describe method of data extraction from reports (e.g., piloted forms, independently, in duplicate) and any processes for obtaining and confirming data from investigators.	6
Definitions for data extraction	11	Provide definitions used in data extraction and classifications of target condition(s), index test(s), reference standard(s) and other characteristics (e.g. study design, clinical setting).	Text S2
Risk of bias and applicability	12	Describe methods used for assessing risk of bias in individual studies and concerns regarding the applicability to the review question.	6
Diagnostic accuracy measures	13	State the principal diagnostic accuracy measure(s) reported (e.g. sensitivity, specificity) and state the unit of assessment (e.g. per-patient, per-lesion).	7

PRISMA-DTA Checklist

Synthesis of results	14	Describe methods of handling data, combining results of studies and describing variability between studies. This could include, but is not limited to: a) handling of multiple definitions of target condition. b) handling of multiple thresholds of test positivity, c) handling multiple index test readers, d) handling of indeterminate test results, e) grouping and comparing tests, f) handling of different reference standards	7-8

PRISMA-DTA Checklist

Adapted From: McInnes MDF, Moher D, Thombs BD, McGrath TA, Bossuyt PM, The PRISMA-DTA Group (2018). Preferred Reporting Items for a Systematic Review and

Section/topic	#	PRISMA-DTA Checklist Item	Reported on page #
Meta-analysis	D2	Report the statistical methods used for meta-analyses, if performed.	7-8
Additional analyses	16	Describe methods of additional analyses (e.g., sensitivity or subgroup analyses, meta-regression), if done, indicating which were pre-specified.	7-8
**RESULTS**			
Study selection	17	Provide numbers of studies screened, assessed for eligibility, included in the review (and included in meta-analysis, if applicable) with reasons for exclusions at each stage, ideally with a flow diagram.	Fig. 1
Study characteristics	18	For each included study provide citations and present key characteristics including: a) participant characteristics (presentation, prior testing), b) clinical setting, c) study design, d) target condition definition, e) index test, f) reference standard, g) sample size, h) funding sources	Table 1
Risk of bias and applicability	19	Present evaluation of risk of bias and concerns regarding applicability for each study.	Figs 2, S1
Results of individual studies	20	For each analysis in each study (e.g. unique combination of index test, reference standard, and positivity threshold) report 2x2 data (TP, FP, FN, TN) with estimates of diagnostic accuracy and confidence intervals, ideally with a forest or receiver operator characteristic (ROC) plot.	Figs 3A-D, S2, 3, 4
Synthesis of results	21	Describe test accuracy, including variability; if meta-analysis was done, include results and confidence intervals.	11-12
Additional analysis	23	Give results of additional analyses, if done (e.g., sensitivity or subgroup analyses, meta-regression; analysis of index test: failure rates, proportion of inconclusive results, adverse events).	11-12 Figs S2-S4
**DISCUSSION**			
Summary of evidence	24	Summarize the main findings including the strength of evidence.	12-13
Limitations	25	Discuss limitations from included studies (e.g. risk of bias and concerns regarding applicability) and from the review process (e.g. incomplete retrieval of identified research).	13-14
Conclusions	26	Provide a general interpretation of the results in the context of other evidence. Discuss implications for future research and clinical practice (e.g. the intended use and clinical role of the index test).	12-14
**FUNDING**			
Funding	27	For the systematic review, describe the sources of funding and other support and the role of the funders.	15

Meta-analysis of Diagnostic Test Accuracy Studies: The PRISMA-DTA Statement. JAMA. 2018 Jan 23;319(4):388-396. doi: 10.1001/jama.2017.19163. For more information, visit: www.prisma-statement.org. (8).

Table S2: PRISMA checklist.

	Risk of bias					Applicability concerns		
	Patient selection	Index test	Reference standard	Flow and timing		Patient selection	Index Test	Reference standard
Banada 2016	-	?	-	+		-	?	?
Chipinduro 2017	?	+	?	?		+	?	?
Hasan 2017	-	+	?	+		+	?	?
LaCourse 2018	+	+	?	+		-	?	?
Marcy 2016	+	+	?	+		-	?	?
Moussa 2016	?	+	?	?		+	?	?
Nicol 2013	+	+	?	+		+	?	?
Orikiriza 2018	+	+	?	?		+	?	?
Walters 2017	+	+	?	+		+	?	?

Figure S1: Risk of bias and applicability concerns summary: review authors' judgements about each domain for each of the nine included studies. A "-" indicates high risk of bias; a "?" indicates unclear risk of bias; a "+" indicates low risk of bias.

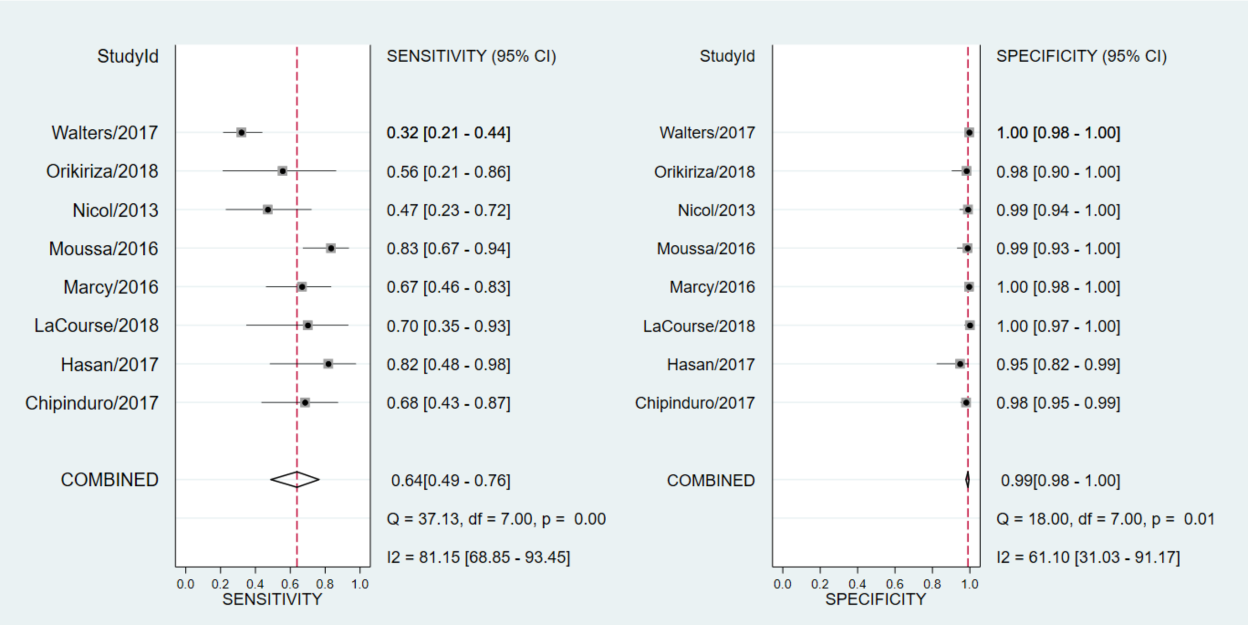

Figure S2a: Forest plots of sensitivity analysis of stool Xpert's diagnostic performance compared to a microbiological reference standard of culture only on respiratory samples using 8 studies; one study (9) that used Xpert as a reference standard was not included here.

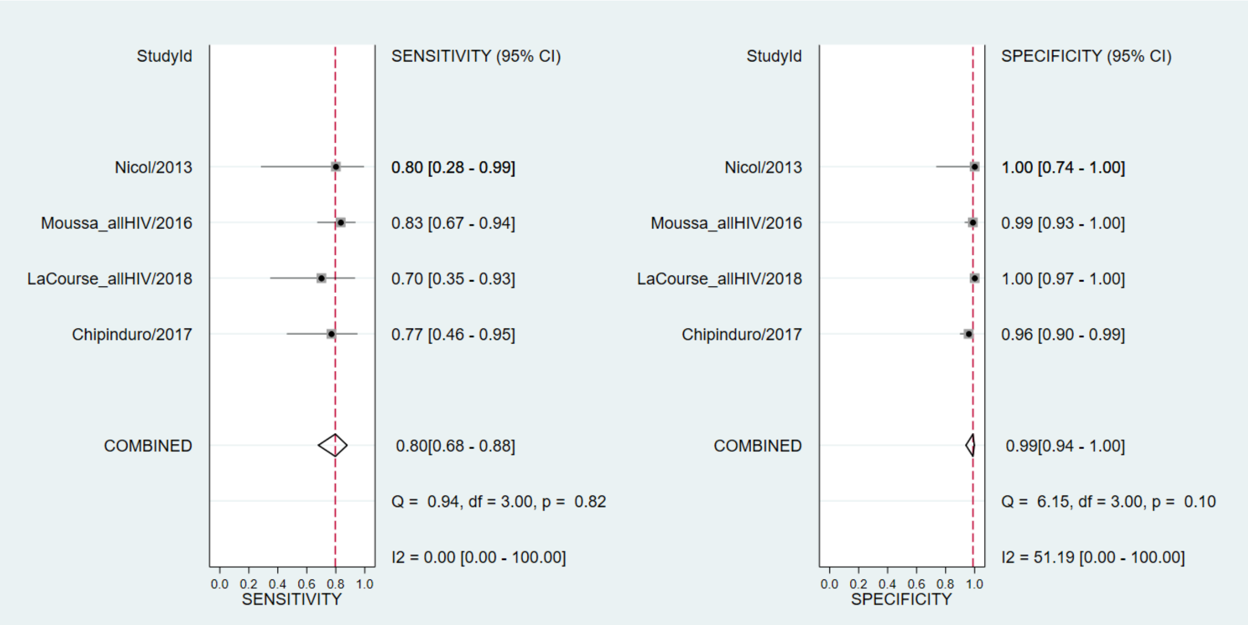

Figure S2b: Forest plots of sensitivity analysis of stool Xpert's diagnostic performance in children living with HIV compared to a microbiological reference standard of culture only on respiratory samples using 8 studies; one study (9) that used Xpert as a reference standard was not included here.

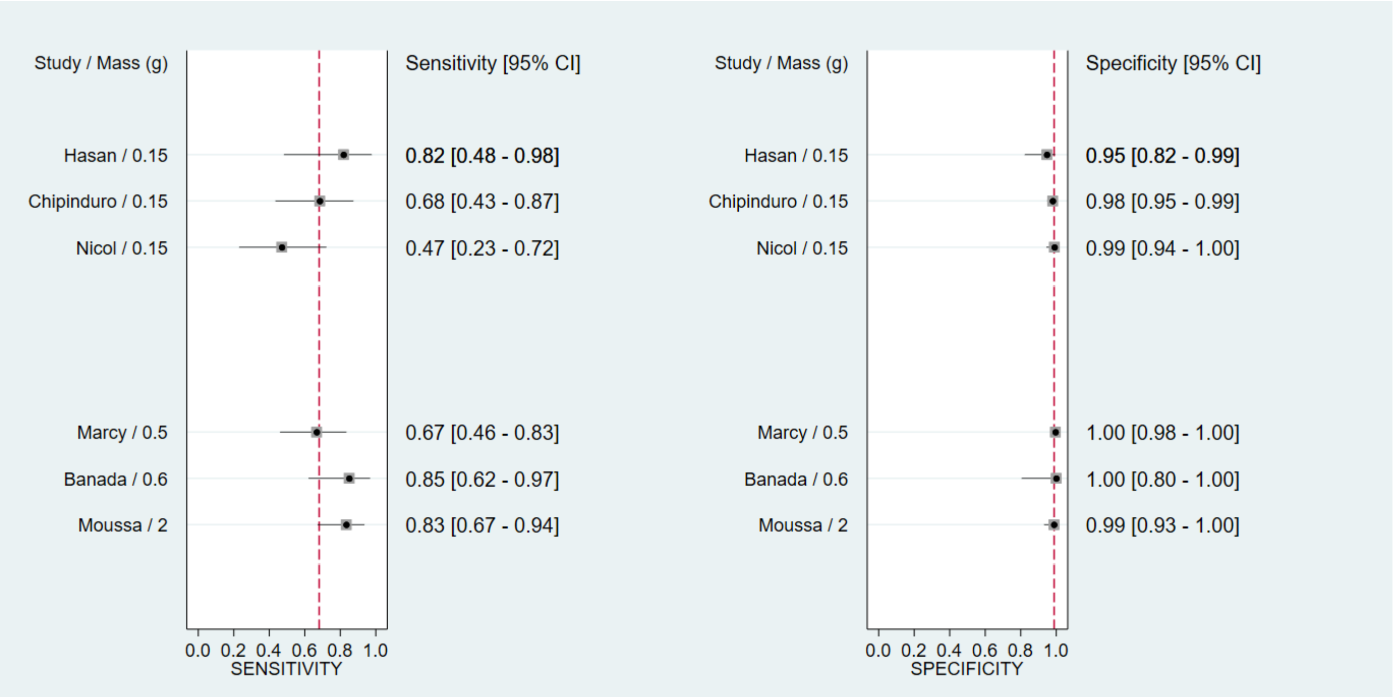

Figure S3: Forest plots of stool Xpert performance arranged by sample mass. The top three listed studies (n=382) utilized 0.15 g of stool collected in bulk, or using a sterile loop or FLOQ swab. The bottom three studies (n=424) used at least 0.5 g of bulk stool.

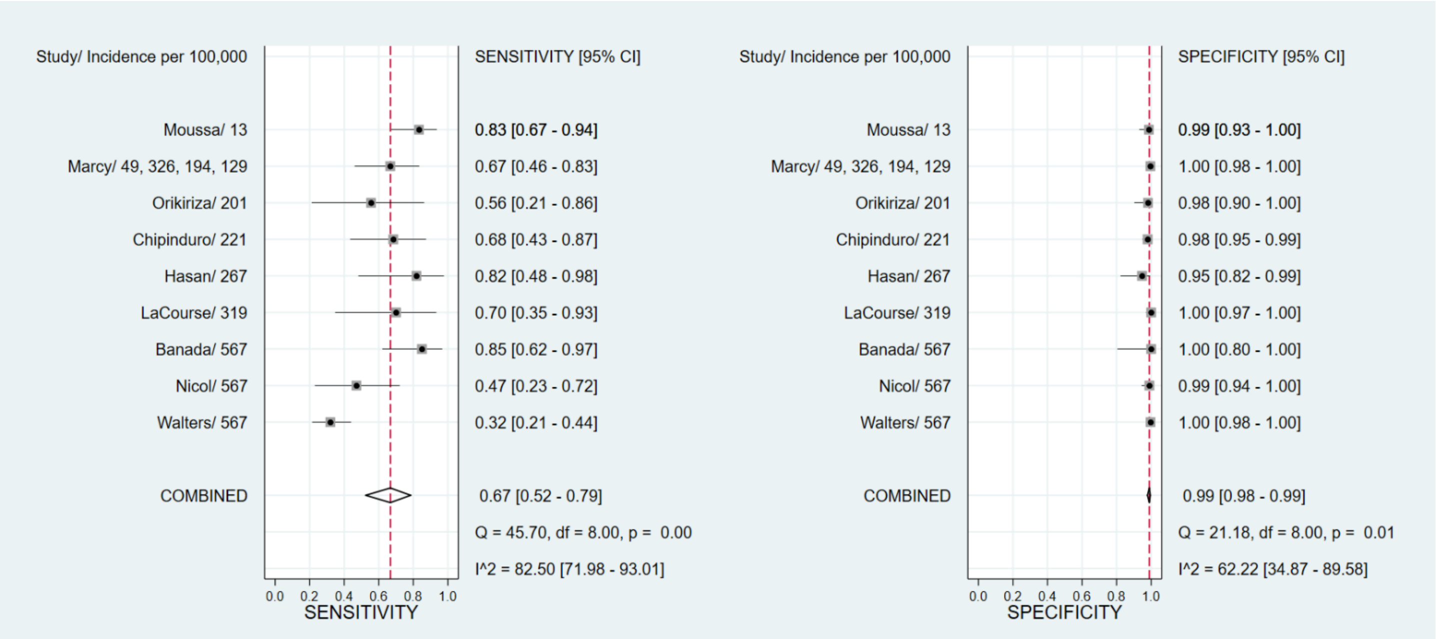

Figure S4: Forest plots of stool Xpert's diagnostic performance compared to a microbiological reference standard, arranged by study countries' TB burdens. Incidence per 100,000 people is presented after the study author. Countries where studies recruited patients are presented in Table 1.

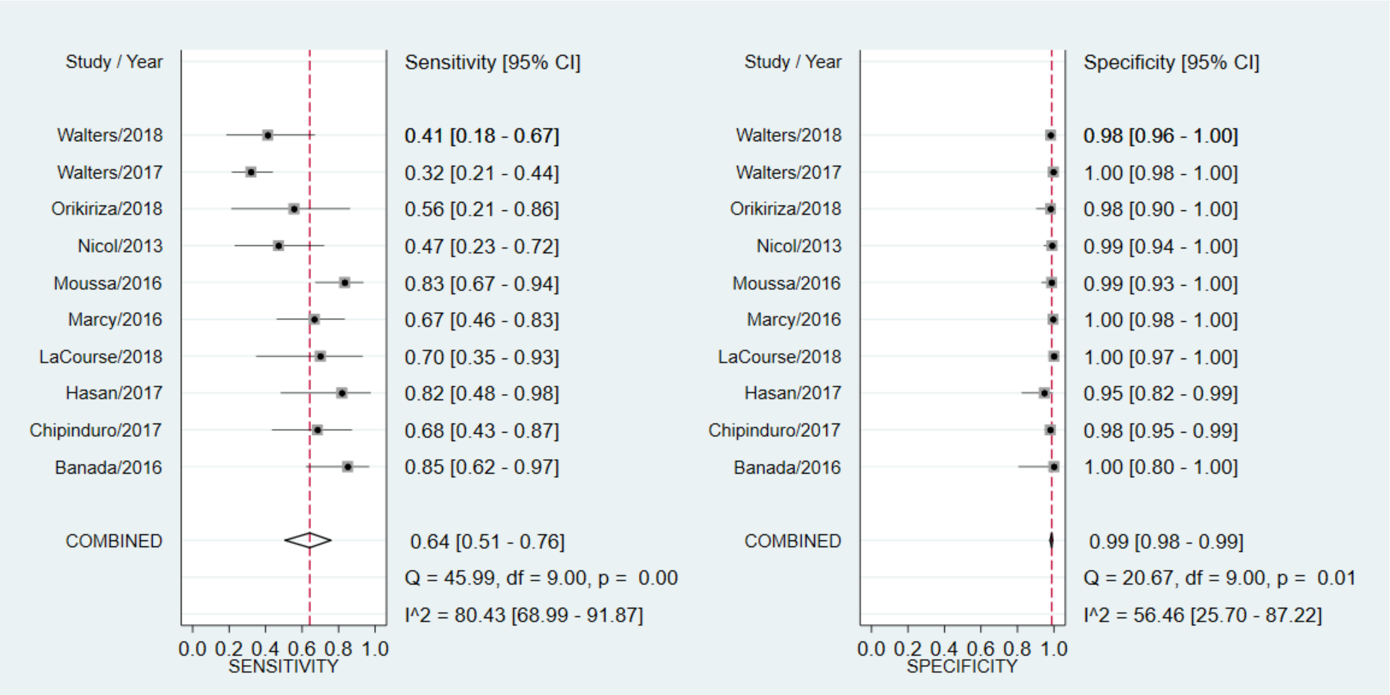

Figure S5: Forest plots of stool Xpert's diagnostic performance compared to a microbiological reference standard of culture or Xpert positivity on respiratory samples using 10 studies, including one study (10) that was published after the date of the systematic search.

Text S1a: Search strategy used for PubMed

No further filters

(((((((Infan* OR newborn* OR new-born* OR perinat* OR neonat* OR baby OR baby* OR babies OR toddler* OR minors OR minors* OR boy OR boys OR boyfriend OR boyhood OR girl* OR kid OR kids OR child OR child* OR children* OR schoolchild* OR schoolchild OR school child[tiab] OR school child*[tiab] OR adolescen* OR juvenil* OR youth* OR teen* OR under*age* OR pubescen* OR pediatrics[mh] OR pediatric* OR paediatric* OR peadiatric* OR school[tiab] OR school*[tiab] OR prematur* OR preterm*)))) AND ((("tuberculosis"[mesh] OR tuberculos*[tw] OR TB[tw] OR "mycobacterium tuberculosis"[mesh]))))) AND (((("Nucleic Acid Amplification Techniques"[Mesh] OR "molecular diagnostic techniques"[mesh:noexp] OR nucleic acid test*[tw] OR NAAT[tw] OR NAATs[tw] OR NAA[tw] OR molecular assay*[tw] OR molecular diagnos*[tw] OR Molecular technique*[tw] OR Molecular test*[tw] OR polymerase chain reaction*[tw] OR PCR[tw] OR PCRs[tw] OR Xpert[tw] OR GeneXpert[tw] OR cepheid[tw] OR MTB/RIF[tw] OR (nucleic acid[tw] AND amplification[tw]) OR NAT[ti] OR NATs[ti] OR (amplified[tw] AND direct test*[tw])))))) AND ("2008/01/01"[PDat] : "2018/06/15"[PDat]))

Text S1b: Search strategy used for EMBASE

Limit date range to 2008-2018, then combine with AND

No other filters

(Infan* or newborn* or new-born* or perinat* or neonat* or baby or baby* or babies or toddler* or minors or minors* or boy or boys or boyfriend or boyhood or girl* or kid or kids or child or child* or children* or schoolchild* or schoolchild or school child or school child* or adolescen* or juvenil* or youth* or teen* or under*age* or pubescen* or pediatrics or pediatric* or paediatric* or peadiatric* or school or school* or prematur* or preterm*).tw

"Nucleic Acid Amplification Techniques".kw. or nucleic acid test*.tw. or NAAT.tw. or NAATs.tw. or NAA.tw. or molecular assay*.tw. or molecular diagnos*.tw. or molecular technique*.tw. or molecular test*.tw. or polymerase chain reaction*.tw. or PCR.tw. or PCRs*.tw. or Xpert.tw. or GeneXpert.tw. or cepheid.tw. or MTB RIF.tw.

("tuberculosis" or tuberculos* or TB or "mycobacterium tuberculosis").tw.

Text S1c: Search strategy used for Scopus

Limit date range to 2008-2018, then combine with AND

No other filters

(TITLE-ABS-KEY ("tuberculosis" OR tuberculos* OR tb OR "mycobacterium tuberculosis") AND PUBYEAR > 2007 AND PUBYEAR < 2019) AND (TITLE-ABS-KEY ("Nucleic Acid Amplification Techniques" OR nucleic AND acid AND test* OR naat OR naats OR naa OR molecular AND assay* OR molecular AND diagnos* OR molecular AND technique* OR molecular AND test* OR polymerase AND chain AND reaction* OR pcr OR pcrs* OR xpert OR genexpert OR cepheid OR mtb) AND PUBYEAR > 2007 AND PUBYEAR < 2018) AND ((TITLE-ABS-KEY (infan* OR newborn* OR new-born* OR perinat* OR neonat* OR baby* OR babies OR toddler* OR minors* OR boy* OR girl* OR kid* OR child OR child* OR children* OR schoolchild* OR adolescen* OR juvenil* OR youth* OR teen* OR pubescen* OR pediatrics OR pediatric*) OR TITLE-ABS-KEY (paediatric* OR prematur* OR preterm*)) AND PUBYEAR > 2007 AND PUBYEAR < 2019)

Text S1d: Search strategy used for Cochrane Library

Title, Abstract, Keywords: tuberculosis AND

Search all text: xpert OR NAAT AND

Search all text: child* OR pediat*

Text S2: Data extracted from each of the 9 studies included in the meta analysis

(i) biomarker: name, biomarker name, number of markers, category of biomarker;
(ii) index test: number of samples tested, place of sample testing, clinical setting, unit of analysis, number of indeterminate results;
(iii) reference standard: reference standard employed and remarks, number of contaminated cultures;
(iv) participant information: descriptive study population information, age demographic, sex, total number of participants, negative population, different populations included in study, HIV status, smear status, EPTB status, other comorbidities, disease contacts, history of TB, symptoms;
(v) study information: author-defined study design, study timing, sampling strategy, study location, study time period, place of sample testing, study location, study time period;
(vi) diagnostic performance data: for (a) microbiological and (b) clinical reference standards, by HIV-status or smear-status or EPTB status if information was available: numbers of true positives, true negatives, false positives, and false negatives, sensitivity and confidence intervals, specificity and confidence intervals, positive and negative predictive values, number of TB cases, number of reference standard negative controls;
(vii) stool processing information;
(viii) bibliographic information

References

1. Chipinduro M, Mateveke K, Makamure B, Ferrand RA, Gomo E. 2017. Stool Xpert(R) MTB/RIF test for the diagnosis of childhood pulmonary tuberculosis at primary clinics in Zimbabwe. Int J Tuberc Lung Dis 21:161-166.

2. Graham SM, Cuevas LE, Jean-Philippe P, Browning R, Casenghi M, Detjen AK, Gnanashanmugam D, Hesseling AC, Kampmann B, Mandalakas A, Marais BJ, Schito M, Spiegel HM, Starke JR, Worrell C, Zar HJ. 2015. Clinical Case Definitions for Classification of Intrathoracic Tuberculosis in Children: An Update. Clin Infect Dis 61Suppl 3:S179-87.

3. Hasan Z, Shakoor S, Arif F, Mehnaz A, Akber A, Haider M, Kanji A, Hasan R. 2017. Evaluation of Xpert MTB/RIF testing for rapid diagnosis of childhood pulmonary tuberculosis in children by Xpert MTB/RIF testing of stool samples in a low resource setting. BMC Res Notes 10:473.

4. National Tuberculosis Control Program. 2015. National guidelines for the management of tuberculosis in Pakistan. Coordination MoNHSR, Pakistan, Islamabad, Pakistan.

5. LaCourse SM, Pavlinac PB, Cranmer LM, Njuguna IN, Mugo C, Gatimu J, Stern J, Walson JL, Maleche-Obimbo E, Oyugi J, Wamalwa D, John-Stewart G. 2018. Stool Xpert MTB/RIF and urine lipoarabinomannan for the diagnosis of tuberculosis in hospitalized HIV-infected children. Aids 32:69-78.

6. Marcy O, Ung V, Goyet S, Borand L, Msellati P, Tejiokem M, Nguyen Thi NL, Nacro B, Cheng S, Eyangoh S, Pham TH, Ouedraogo AS, Tarantola A, Godreuil S, Blanche S, Delacourt C. 2016. Performance of Xpert MTB/RIF and Alternative Specimen Collection Methods for the Diagnosis of Tuberculosis in HIV-Infected Children. Clinical Infectious Diseases 62:1161-1168.

7. Walters E, Van Der Zalm MM, Palmer M, Bosch C, Demers AM, Draper H, Goussard P, Schaaf HS, Friedrich SO, Whitelaw A, Warren R, Gie RP, Hesseling AC. 2017. Xpert MTB/RIF on Stool Is Useful for the Rapid Diagnosis of Tuberculosis in Young Children with Severe Pulmonary Disease. Pediatric Infectious Disease Journal 36:837-843.

8. McInnes MDF, Moher D, Thombs BD, McGrath TA, Bossuyt PM, Clifford T, Cohen JF, Deeks JJ, Gatsonis C, Hooft L, Hunt HA, Hyde CJ, Korevaar DA, Leeflang MMG, Macaskill P, Reitsma JB, Rodin R, Rutjes AWS, Salameh JP, Stevens A, Takwoingi Y, Tonelli M, Weeks L, Whiting P, Willis BH. 2018. Preferred Reporting Items for a Systematic Review and Meta-analysis of Diagnostic Test Accuracy Studies: The PRISMA-DTA Statement. Jama 319:388-396.

9. Banada PP, Naidoo U, Deshpande S, Karim F, Flynn JL, O'Malley M, Jones M, Nanassy O, Jeena P, Alland D. 2016. A novel sample processing method for rapid detection of tuberculosis in the stool of pediatric patients using the Xpert MTB/RIF assay. PLoS ONE 11 (3) (no pagination).

10. Walters E, Scott L, Nabeta P, Demers AM, Reubenson G, Bosch C, David A, van der Zalm M, Havumaki J, Palmer M, Hesseling AC, Ncayiyana J, Stevens W, Alland D, Denkinger C, Banada P. 2018. Molecular detection of Mycobacterium tuberculosis from stool in young children using a novel centrifugation-free processing method. J Clin Microbiol doi:10.1128/jcm.00781-18.
